# MicroRNA-221 Modulates RSV Replication in Human Bronchial Epithelium by Targeting NGF Expression

**DOI:** 10.1371/journal.pone.0030030

**Published:** 2012-01-17

**Authors:** Sreekumar Othumpangat, Cheryl Walton, Giovanni Piedimonte

**Affiliations:** Department of Pediatrics and Pediatric Research Institute, West Virginia University School of Medicine, Morgantown, West Virginia, United States of America; Johns Hopkins University - Bloomberg School of Public Health, United States of America

## Abstract

**Background:**

Early-life infection by respiratory syncytial virus (RSV) is associated with aberrant expression of the prototypical neurotrophin nerve growth factor (NGF) and its cognate receptors in human bronchial epithelium. However, the chain of events leading to this outcome, and its functional implications for the progression of the viral infection, has not been elucidated. This study sought to test the hypothesis that RSV infection modulates neurotrophic pathways in human airways by silencing the expression of specific microRNAs (miRNAs), and that this effect favors viral growth by interfering with programmed death of infected cells.

**Methodology:**

Human bronchial epithelial cells infected with green fluorescent protein-expressing RSV (rgRSV) were screened with multiplex qPCR arrays, and miRNAs significantly affected by the virus were analyzed for homology with mRNAs encoding neurotrophic factors or receptors. Mimic sequences of selected miRNAs were transfected into non-infected bronchial cells to confirm the role of each of them in regulating neurotrophins expression at the gene and protein level, and to study their influence on cell cycle and viral replication.

**Principal Findings:**

RSV caused downregulation of 24 miRNAs and upregulation of 2 (p<0.01). Homology analysis of microarray data revealed that 6 of those miRNAs exhibited a high degree of complementarity to NGF and/or one of its cognate receptors TrKA and p75^NTR^. Among the selected miRNAs, miR-221 was significantly downregulated by RSV and its transfection in bronchial epithelial cells maximally inhibited gene and protein expression of NGF and TrKA, increased apoptotic cell death, and reduced viral replication and infectivity.

**Conclusions/Significance:**

Our data suggest that RSV upregulates the NGF-TrKA axis in human airways by silencing miR-221 expression, and this favors viral replication by interfering with the apoptotic death of infected cells. Consequently, the targeted delivery of exogenous miRNAs to the airways may provide a new strategy for future antiviral therapies based on RNA interference.

## Introduction

Respiratory syncytial virus (RSV) infection modifies the expression of critical neurotrophic factors and receptors [1], and this effect has important implications for the pathophysiology of airway inflammation and hyperreactivity during and after the acute infection [2]. In particular, we have shown previously a significant upregulation of the nerve growth factor (NGF) and its cognate high-affinity receptor tropomyosin-related kinase A (TrkA), with concomitant downregulation of the low-affinity pan-neurotrophin p75^NTR^ receptor [3]. As NGF prevents apoptosis through TrkA by increasing expression of the anti-apoptotic Bcl-2 family members, whereas p75^NTR^ signaling promotes apoptosis via JNK, NF-κB and ceramide activation, these adjustments are geared to keep the infected airway epithelial cells alive to support RSV replication and infection with a mechanism similar to that previously described in macrophages infected with the human immunodeficiency virus (HIV) [4].

We have confirmed these interactions between RSV and neurotrophic pathways in humans, showing increased NGF and TrkA expression in the cell fractions obtained by bronchoalveolar lavage from infants with active RSV infection [5], and we have also localized these changes to the epithelium of the distal airways [3]. However, the exact mechanisms involved in the upregulation of NGF expression by RSV and its functional implications for the efficiency and progression of the infection are still poorly understood, and additional knowledge in this area could result in significant progress toward the therapy of this highly prevalent disease.

The discovery of RNA interference [6] has broadened dramatically our understanding of the epigenetic mechanisms regulating gene expression, and considerable attention has been dedicated to the role of microRNA (miRNA) species [7]. Mature miRNAs are non-coding transcripts 18 to 25 nucleotides in length that modulate protein expression post-transcriptionally, by binding to complementary or partially complementary messenger RNA (mRNA) targets and priming them for degradation or translational inhibition. In humans, the extensive regulatory network involving >700 miRNAs, each able to target >300 different mRNAs, is predicted to regulate >60% of coding genes [8] and has been implicated in a wide range of fundamental cellular processes including proliferation, differentiation, and death [9]. Furthermore, >200 miRNAs encoded by the genome of several families of mammalian viruses have been identified.

In the present study, we tested the hypothesis that specific miRNA species modulate the expression of key neurotrophic factor and receptors in human bronchial epithelial cells, and are in particular responsible for the dysregulation of neurotrophic pathways during RSV infection. For this, human bronchial epithelial cells infected with green fluorescent protein (GFP)-expressing recombinant RSV (rgRSV) were screened with miRNA microarrays, and homology search identified specific miRNA species complimentary to NGF or its cognate receptors TrKA and p75^NTR^. Mimic sequences of the selected miRNAs were then transfected into non-infected bronchial cells to confirm their role in the regulation of neurotrophin expression using quantitative real-time polymerase chain reaction (qPCR) and fluorescence activated cell sorting (FACS). This explorative analysis allowed to focus our attention on miR-221 for more mechanistic experiments involving FACS and confocal microscopy to study the influence of this miRNA on NGF expression and programmed death of infected cells. Finally, we tested the hypothesis that synthetic homologues of miR-221 delivered directly to the bronchial epithelium affect RSV replication, and may therefore have therapeutic potential against this infection.

## Methods

### Cells and virus

Human bronchial epithelial cells derived from the surface epithelium of normal Caucasian or Black donors of both sexes with ages ranging from 16 to 65 years were purchased from Cell Applications (San Diego, CA) and were maintained at 37°C in the proprietary growth media and supplements provided by the vendor [3]. Each experiment was repeated using cells from different donors throughout the study to control for host genetics and environment.

The rgRSV stock derived from the RSV-A_2_ strain and expressing the green fluorescent protein gene [10] was kindly provided by Dr. Mark Peeples (Columbus Children Research Institute, Columbus, OH) and Dr. Peter Collins (National Institutes of Health, Bethesda, MD). Cultivation and harvesting of rgRSV was performed at West Virginia University as described previously [3]. To study miRNA expression patterns during infection, the bronchial cells were grown in 6-well plates for 48 h and subsequently infected for 24 h with rgRSV at multiplicity of infection (MOI) of 1. Duration and multiplicity of infection were chosen based on previously published studies [3]. Also, we did not run control experiments with UV-inactivated virus because our previous studies have shown that UV-RSV has no effect on neurotrophins expression in airway epithelial cells [3].

### miRNA analysis

miRNA expression in rgRSV-infected bronchial epithelial cells and in non-infected controls was compared using the *mir*Vana™ isolation kit (Ambion, Austin, TX) and the TaqMan® MicroRNA assay (Applied Biosystems, Foster City, CA). This multiplex qPCR array is based on 384-well microfluidic cards [TaqMan® Low-Density Array (TLDA)] with primers and probe for each assay preloaded and dried onto the designated duplicate wells [11], which enables the accurate quantitation of 378 mature human miRNAs. Mammalian U6 (MammU6), RNU44, and RNU48 were used as endogenous housekeeping genes to aid in data normalization. Data sets generated by Genome Explorations (Memphis, TN) were analyzed with the software SDS version 2.3 and AB qPCR-R (Applied Biosystems). We used TargetScan and miRBase as bioinformatics tools for target identification.

### Transfection

Bronchial epithelial cells were reverse-transfected with precursor (pre)-miRNA oligonucleotides using the lipid-based siPORT *NeoFX*™ reagent diluted in Opti-MEM® I reduced serum medium (Gibco, Grand Island, NY) according to the protocol provided by the manufacturer (Ambion). Briefly, cells in their early passages (passage 2 or 3) were trypsinized and counted. Transfection complexes were directly applied to the cells (50,000 cells/well in 6-well plates) to reach a final pre-miRNA oligonucleotide concentration of 50 nM, and the plates were incubated in a humidified chamber with 5% CO_2_ at 37°C for 24 h. Control cells were transfected with the Pre-miR™ negative control provided by the same vendor. To study the effects of selected miRNAs in the context of viral infection, cells surviving 48 h after transfection were incubated with rgRSV at MOI of 1. After 6 h of infection, the excess virus was washed off and fresh growth medium was added. Cells were then incubated for another 18 h (i.e., a total of 72 h after starting the transfection). At the end of the incubation, cells were harvested by trypsinization, washed with PBS, and used for further analysis.

### qPCR

Total RNA was isolated from bronchial epithelial cells using the RNeasy kit (Qiagen, Valencia, CA) and 100 ng per reaction was used for qPCR analysis using the SYBR green one-step RT-PCR master mix (Qiagen) and the ABI 7500 real-time cycler (Applied Biosystems). NGF, TrKA, and p75^NTR^ primers were purchased from SABiosciences (Rockville, MD). Transcript expression was normalized using hypoxanthine phosphoribosyltransferase 1 (HPRT1; RealTimePrimers, Elkins Park, PA) as the housekeeping gene. Relative change in gene expression was calculated using the formula: fold change = 2∧^−(ddCt)^ = 2-dCt (treated samples)−dCt (control samples), where dCt = Ct (detected gene)−Ct (HPRT1) and Ct is the threshold number. Cycle threshold (C_t_) values were calculated with the SDS software using automatic baseline settings with assigned minimum threshold of 0.2. Because the C_t_ value of 35 represents single template detection, C_t_ values >35 were considered to be below the detection level of the assay.

### FACS

Bronchial epithelial cells were isolated by trypsinization and stained with specific antibodies for intracellular and surface detection of NGF, TrKA, and p75^NTR^ (Santa Cruz Biotechnology, Santa Cruz, CA). Rabbit IgG (Southern Biotechnology, Birmingham, AL) was used as isotype control for non-specific binding. Secondary antibodies were labeled with Alexa 488 and Alexa 546 (Invitrogen, Carlsbad, CA). Data were acquired using a FACSCalibur instrument (BD Biosciences, Franklin Lakes, NJ) and analyzed with the software Windows Multiple Document Interface version 2.9 for flow cytometry (WinMDI, La Jolla, CA).

To detect rgRSV infection, the cells were trypsinized, fixed with 1% formaldehyde for 20 min, centrifuged and resuspended in PBS, and analyzed using FACSCalibur. A minimum of 10,000 events were collected per sample. Non-infected cells were used to establish the background level of fluorescence in the FL-1 channel (488 nm for excitation and 515–545 nm for GFP detection). Once background fluorescence was established, a marker was drawn such that <1% of non-infected cells were considered positive. Mean fluorescent intensity was calculated using the WinMDI software.

### Cell death

Non-infected and rgRSV-infected bronchial epithelial cells were transfected with 50 nM of pre-miR-221 or with the negative control miRNA. Cells were harvested 48 h later, resuspended in 100 µl of 1× annexin binding buffer, and stained for 10 min with 5 µl of annexin V-PE (MBL International, Woburn, MA) and 5 µl of propidium iodide (PI; BD Biosciences, San Jose, CA) to detect apoptosis and necrosis, respectively. After adding 400 µl of 1× buffer to each sample, flow cytometry analysis was performed using a FACSCalibur instrument.

### Virus titration

rgRSV titration was performed as described previously [12]. Briefly, serial dilutions of each sample were prepared in culture medium and 50 µl of the each dilution was inoculated in duplicate on sterile 96-well plates of bronchial cells. After 24 h incubation, the cells were observed under a fluorescent microscope for green fluorescence (EVOS, Advanced Microscopy Group, Bothell, WA). Virus titer was calculated using the following formula: (number of infectious units)×(1/dilution)/(volume of inoculum). Since this assay tests for infection and production of GFP rather than virus spread and plaque formation, this infectivity assay is more accurate than a titer determined by plaque assay.

### Confocal microscopy

As described previously [3], cells transfected with the precursor of miR-221 or with the negative control miRNA were stained for 1 h with rabbit anti-human NGF antibody (Santa Cruz Biotechnology), followed by a secondary PE-conjugated donkey anti-rabbit antibody (Invitrogen) for another hour, and then were incubated with rgRSV at MOI of 1 for 24 h. The nuclei were stained with TOPRO-3 (Invitrogen). The glass slides were mounted with Prolong Gold anti-fade reagent (Invitrogen) and protected with cover slips. Images were obtained using a Zeiss LSM510 confocal microscope with the AxioImager Z1 system (Carl Zeiss, Jena, Germany).

### Statistical analysis

All data are expressed as the mean ± SEM. Data from qPCR experiments are the average of 3 or 4 independent assays performed in duplicate. False discovery rate (FDR)-corrected p-values were calculated with the Benjamini-Hochberg method, and were all <0.1 [13]. Geometric mean fluorescent intensity (MFI) was averaged from the flow cytometry measurements obtained in 3 or 4 independent experiments. The Kruskal-Wallis one-way analysis of variance was used to analyze the effects of different miRNAs on neurotrophins expression, and post-hoc pairwise multiple comparisons between means were performed using the Student-Newman-Keuls method with the statistical significance threshold set at p<0.01. The paired Student's *t* test was used to analyze differences between rgRSV-infected cells transfected with miR-221 vs. negative control miRNA. Statistical analysis was performed using the software SigmaStat version 3.5 for Windows (Systat Software, Point Richmond, CA).

## Results

**Figure 1:**
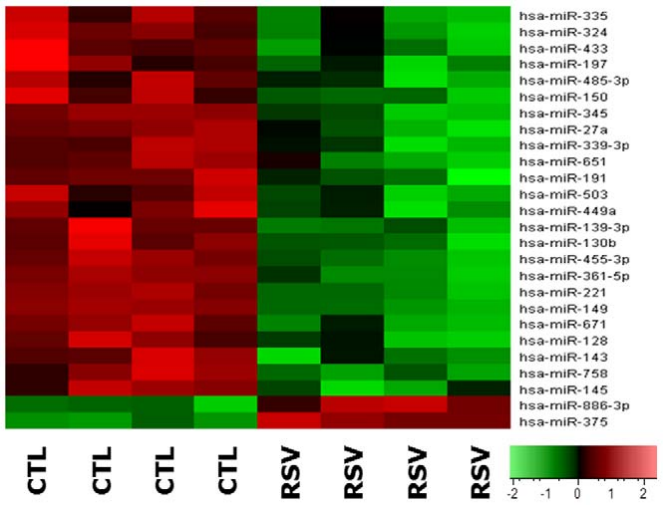
RSV-induced change in microRNAs expression. This heat map was generated using differentially expressed genes based on unadjusted t-test p-values, and provides an overall picture of the impact of RSV infection on the human bronchial epithelial cell miRNAome as compared to non-infected control cells. Data were partitioned between RSV and control (CTL) samples during the hierarchical clustering and organized into blocks of columns. The rows represent wells in each of the plates, and within each row the green shaded areas indicate lower expression of the specific miRNA noted on the right, whereas the red shaded areas indicate higher expression. Black and darkly shaded areas indicate relatively similar expression between infected and non-infected cells. The Euclidean clustering method was used for array data analysis, and the cutoff level of statistical significance was set at p<0.01 to reduce clutter. The expression index is shown in the right lower corner.

Microarray analysis of human bronchial epithelial cells detected significant changes in the global profile of miRNA expression after infection with rgRSV. Specifically, at the p<0.05 level of statistical significance 104 miRNAs were differentially expressed between RSV-infected cells and non-infected controls, of which 101 were significantly downregulated and 3 upregulated. At the p<0.01 level of statistical significance, 24 miRNAs were downregulated and 2 miRNAs were upregulated. The heat map in Figure 1 provides an overall picture of the impact of RSV infection on the human bronchial epithelial cell miRNAome as compared to non-infected control cells, with the cutoff level of statistical significance set at p<0.01 in order to reduce clutter.

From this dataset, we identified 6 *Homo sapiens* (hsa)-miRNAs targets that were significantly affected by RSV and were also holding a high degree of homology to the mRNA sequences encoding neurotrophic factors or receptors: miR-27a, miR-221, miR-339-5p, miR-453, miR-574, and miR-744 (Table 1). We also included in our analysis the maximally downregulated miR-331-5p, even though it did not appear to be involved directly in the expression of any neurotrophin. Mimic oligonucleotides of the precursors for each of these miRNAs were transfected into non-infected human bronchial epithelial cells to screen their individual role in the modulation of neurotrophins expression at the gene and protein level.

**Table 1:** RSV-induced Changes in the Expression of MicroRNAs Predicted to Target Neurotrophic Pathways.

MicroRNA	Relevant Target Gene	Relevant Target Function	Fold Change	*P*-value
**hsa-miR-331-5p**	AIP1	Apoptosis	−3.805	<0.05
**hsa-miR-744**	NGF receptor	NGF binding	3.02	<0.05
**hsa-miR-453**	TrKA, p75^NTR^	NGF binding	−2.012	<0.05
**hsa-miR-221**	ITGB3, NGF, NGF receptor	NGF expression and binding	−1.99	<0.01
**hsa-miR-27a**	p75^NTR^, VEGF-C	NGF binding, angiogenesis	−1.65	<0.01
**hsa-miR-339-5p**	TrKB, APAF1	TrKB-BDNF binding, apoptosis	−1.093	<0.01
**hsa-miR-574**	NGF	NGF gene expression	−0.5	<0.05

***Definition of abbreviations***: hsa: *Homo sapiens*; AIP1: apoptosis inducing protein 1; NGF: nerve growth factor; TrK: tropomyosin-related kinase; ITGB3: integrin beta 3; VEGF-C: vascular endothelial growth factor C; APAF1: apoptotic peptidase activating factor 1; BDNF: brain-derived neurotrophic factor.

***Note***: Negative values represent downregulation.

**Figure 2:**
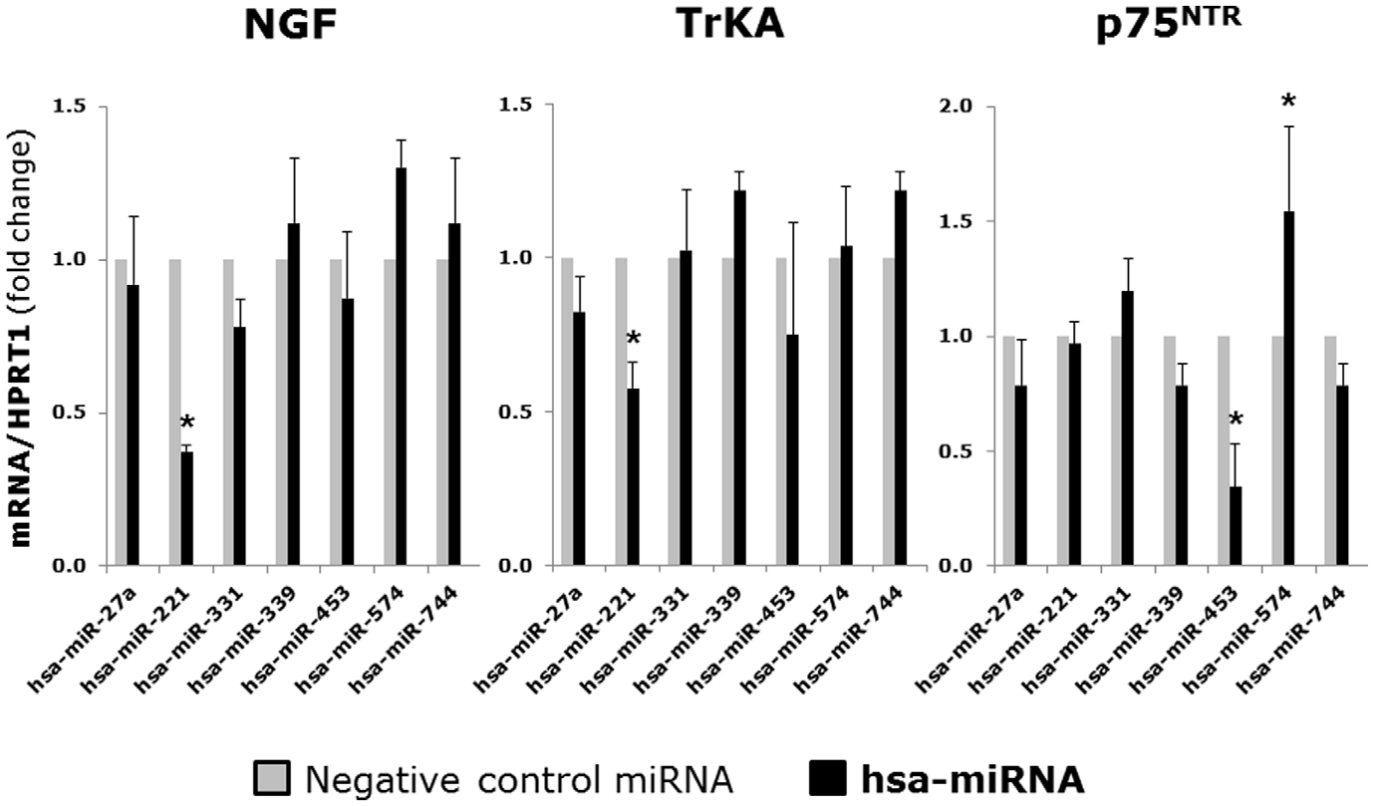
Gene expression in bronchial cells transfected with microRNAs. *Homo sapiens* (hsa)-miRNAs with a high degree of homology to mRNAs encoding neurotrophic factors or receptors were selected from our microarray dataset, and mimic oligonucleotides of the precursors for each of these miRNAs were transfected into human bronchial epithelial cells to study their individual role in modulating neurotrophin expression. PCR measured maximally reduced concentrations of both NGF and TrKA transcripts in bronchial epithelial cells overexpressing miR-221. Data are expressed as the mean ± SEM (n = 3–4 per group). * = p<0.01 compared to cells transfected with the negative control miRNA.

**Figure 3:**
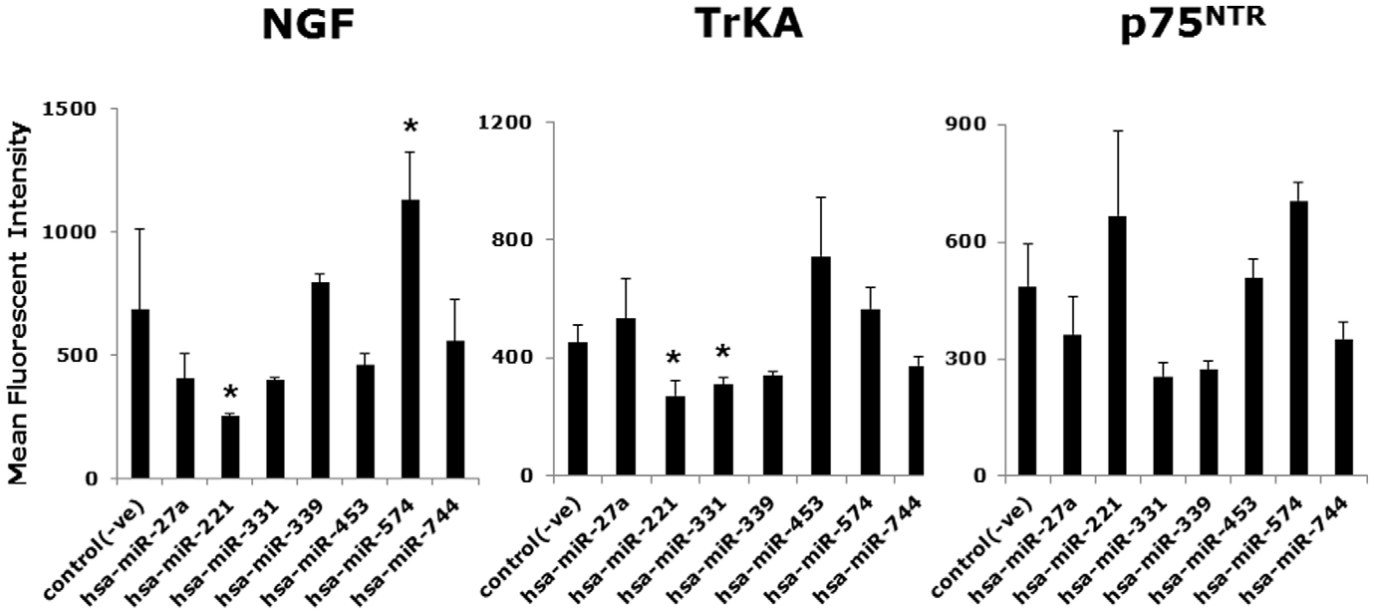
Flow cytometry of bronchial cells transfected with microRNAs. Consistent with the gene expression data, miR-221 transfection was associated to maximal downregulation of NGF protein synthesis, and was also associated to maximal downregulation of its high-affinity TrKA receptor. Data are expressed as the mean ± SEM. Geometric mean fluorescent intensity (MFI) was averaged from the flow cytometry measurements obtained in 3 or 4 independent experiments. * = p<0.01 compared to cells transfected with the negative control miRNA [control(-ve)].

Gene expression analysis of non-infected bronchial cells by qPCR (Figure 2) showed maximal downregulation of both NGF and its high-affinity TrKA receptor in cells overexpressing miR-221. In contrast, the low-affinity p75^NTR^ receptor gene was not affected by miR-221, but was downregulated by miR-453 and upregulated by miR-574. Flow cytometry analysis (Figure 3) confirmed the anti-neurotrophic activity of miR-221, as non-infected bronchial epithelial cells overexpressing this miRNA had maximally reduced levels of both NGF and TrKA protein compared to those transfected with the negative control miRNA. Also, NGF protein was upregulated by miR-574 and TrKA was downregulated by miR-331.

**Figure 4:**
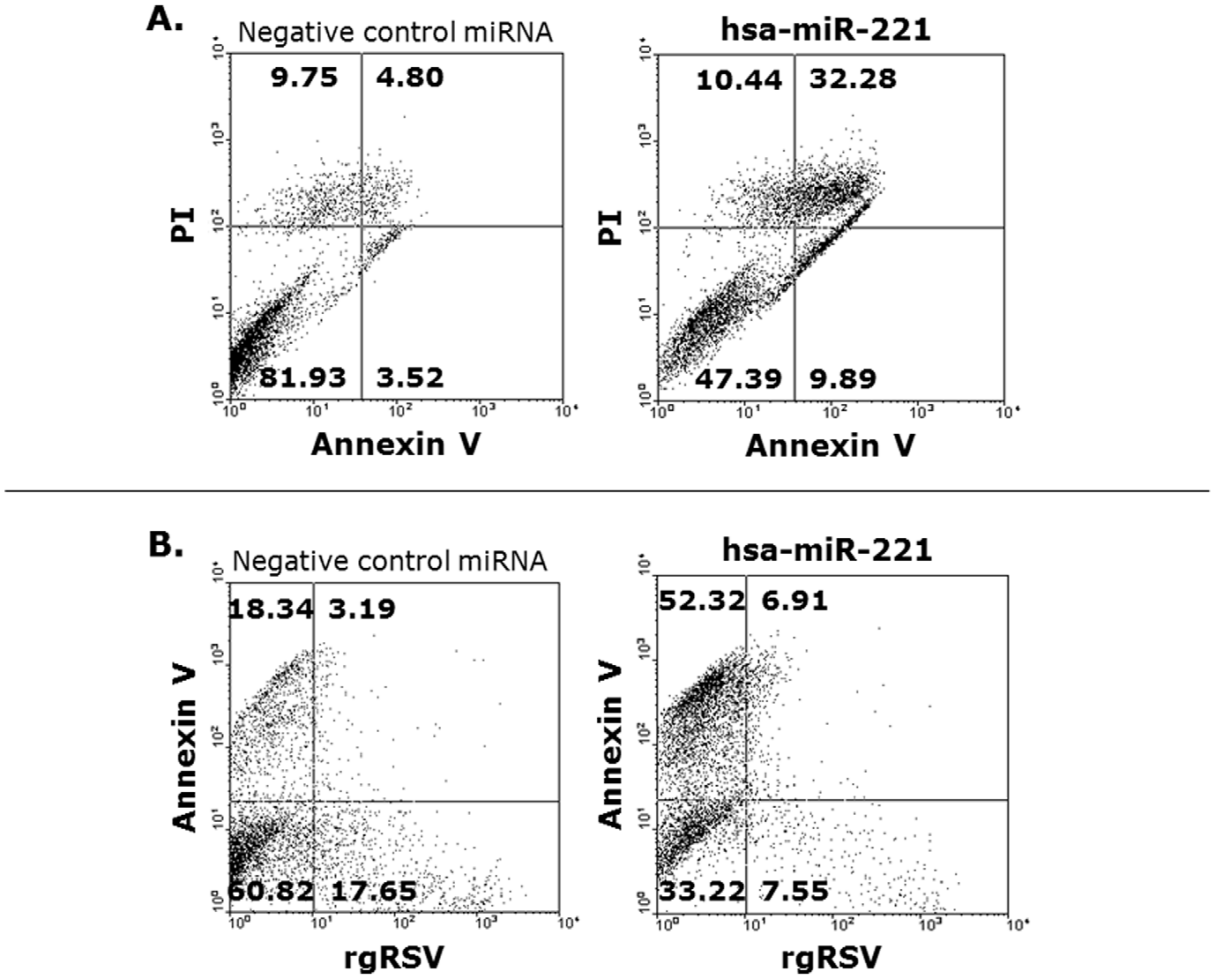
Programmed death of RSV-infected cells. (A) Non-infected cells transfected with hsa-miR-221 were more prone to apoptosis detected by annexin V staining compared to cells transfected with the negative control miRNA. In contrast, hsa-miR-221 transfection did not change the proportion of necrotic cells detected by propidium iodide (PI) staining. (B) After infection with rgRSV, approximately 3 times more hsa-miR-221-transfected cells became apoptotic compared to cells transfected with negative control miRNA. Left upper quadrant = necrotic cells; left lower quadrant = viable cells; right lower quadrant = apoptotic cells; right upper quadrant = late apoptotic cells in necrotic state.

Based on the combined analysis of qPCR and FACS data, we focused our attention on miR-221 to further study the role of the NGF-TrKA axis in the regulation of programmed cell death and viral replication. Cells overexpressing miR-221 were characterized by important changes in programmed death. Non-infected cells transfected with miR-221 (Figure 4A) were more prone to apoptosis (43%) compared to cells transfected with the negative control miRNA (8%). Furthermore, only 47% of the miR-221-overexpressing cells survived, compared to 82% of cells transfected with the negative control. In contrast, the proportion of necrotic cells did not change after transfection with miRNA-221 compared to the negative control (10.4 vs. 9.8). More importantly, 59% of miR-221-transfected cells became apoptotic after infection with rgRSV (Figure 4B), compared to only 22% of the infected cells transfected with negative control miRNA.

**Figure 5:**
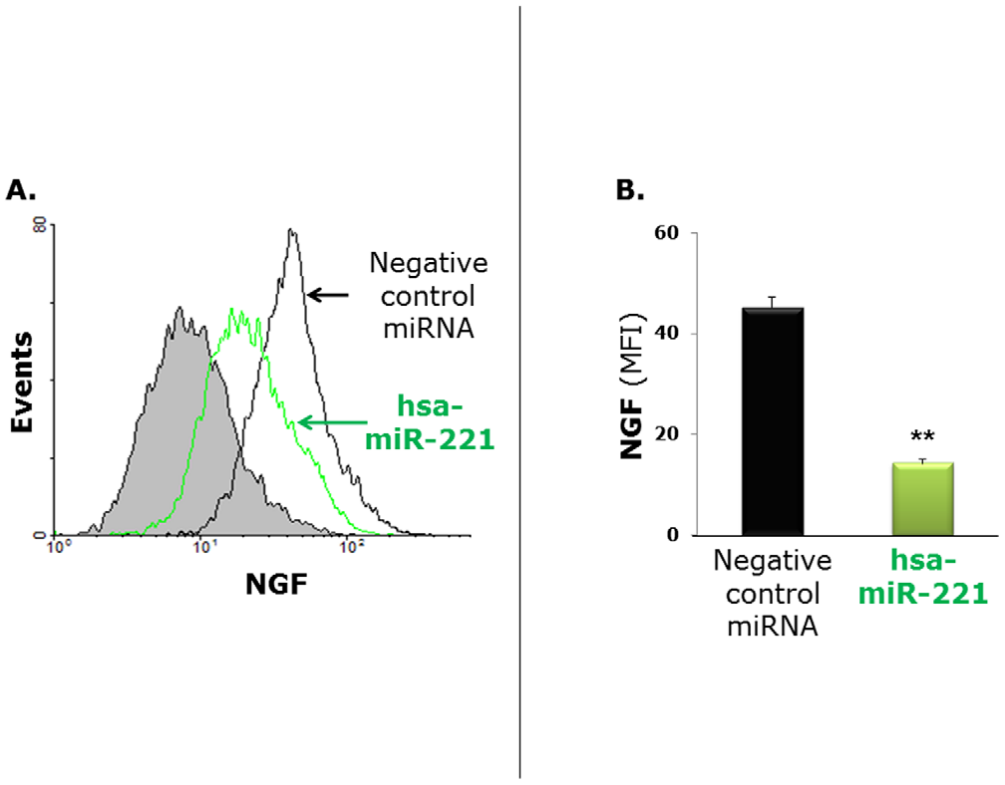
Combined effect of miR-221 and RSV on NGF expression. (A) Flow cytometry analysis of hsa-miR-221-overexpressing bronchial epithelial cells 24 h after infection with rgRSV showed significantly lower expression of NGF protein compared to infected cells with normal miR-221 expression. The gray population represents the isotype control. (B) Geometric mean fluorescent intensity (MFI) was averaged from the flow cytometry measurements obtained in 3 or 4 independent experiments. Data are expressed as the mean ± SEM. * = p<0.01 compared to cells transfected with the negative control miRNA.

**Figure 6:**
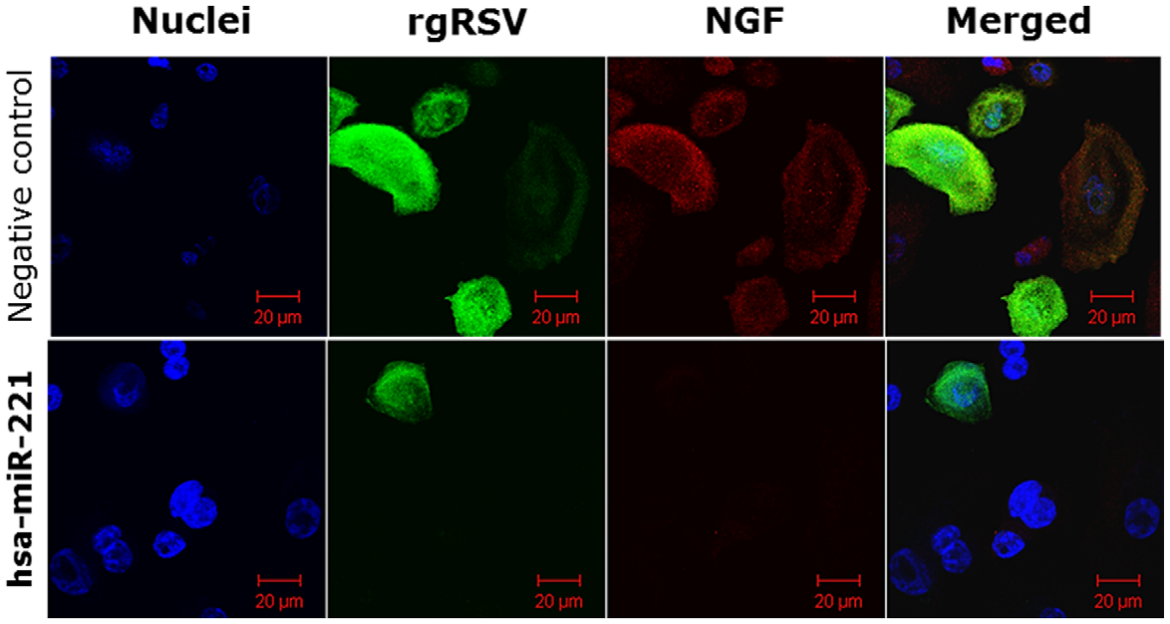
Confocal microscopy of RSV-infected cells. After 24 h infection with rgRSV, most of the bronchial epithelial cells transfected with negative control miRNA (top panels) showed strong green fluorescence as a result of active rgRSV infection and concomitant red fluorescence upregulation of NGF expression. In contrast, NGF expression was silenced in bronchial epithelial cells overexpressing hsa-miR-221 (bottom panels), and this effect was associated with a sharp reduction in the number of cells displaying green fluorescence. Bar scale = 20 µm.

Flow cytometry analysis of bronchial epithelial cells transfected with miR-221 and then infected for 24 h with rgRSV at MOI of 1 (Figure 5) confirmed a significantly lower NGF protein expression compared to rgRSV-infected cells transfected with the negative control miRNA. The combined effect of miR-221 on NGF expression and RSV replication was visualized by confocal microscopy of bronchial epithelial cells incubated for 24 h with rgRSV (Figure 6). Most of the bronchial epithelial cells transfected with negative control miRNA showed strong green fluorescence as a result of active rgRSV infection overlapping the red fluorescence generated by concomitant overexpression of PE-labeled NGF. In contrast, NGF expression was silenced in bronchial cells transfected with miR-221, and this was associated with a sharp reduction in the proportion of infected cells.

**Figure 7:**
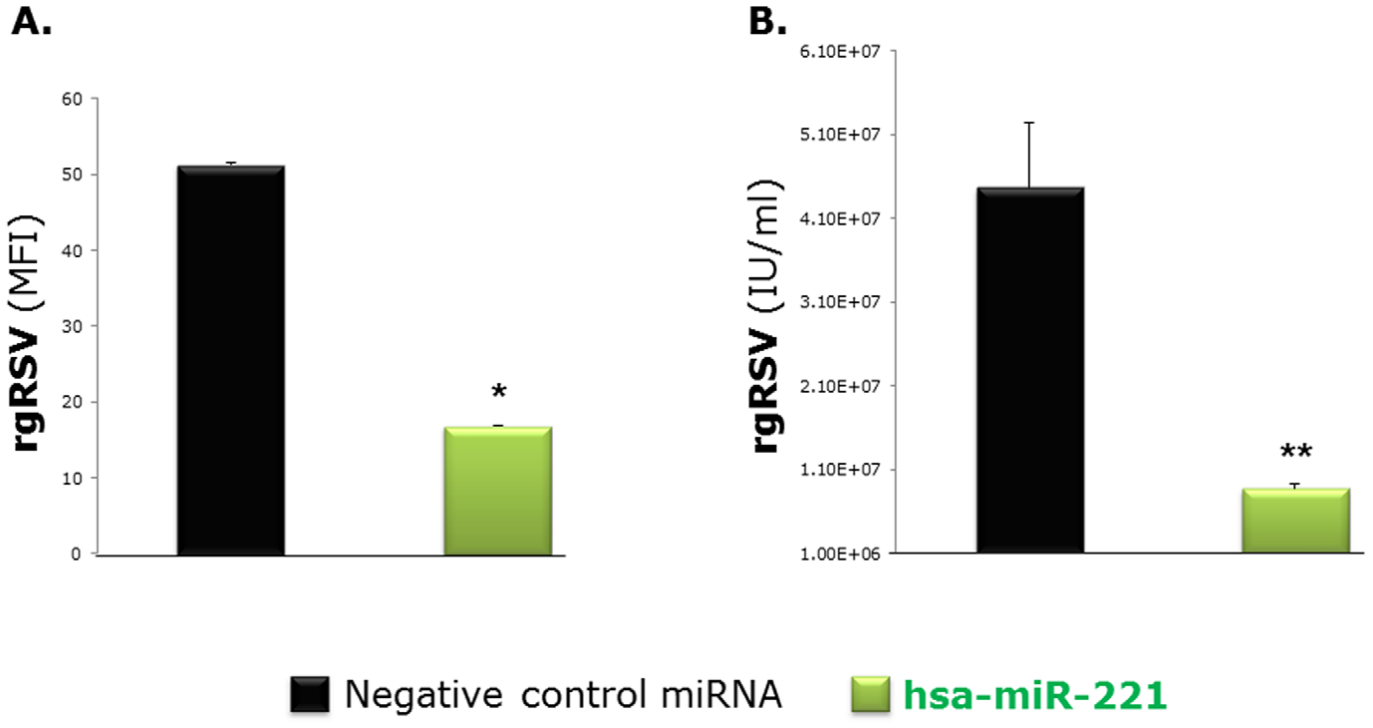
Anti-viral effect of miR-221. (A) FACS showed that hsa-miR-221 transfection reduces significantly the number of rgRSV-infected bronchial cells compared to control cells with normal hsa-miR-221 expression. Geometric mean fluorescent intensity (MFI) was averaged from the flow cytometry measurements obtained in 3 independent experiments. (B) Even stronger inhibition was measured in separate experiments by the titration of fluorescent virus infectious units per ml (IU/ml; n = 6 per group). Data are expressed as the mean ± SEM. ** = p<0.01 compared to cells transfected with the negative control miRNA.

Consistently, the number of rgRSV-infected bronchial cells counted by FACS was reduced strongly by the transfection of miR-221 (Figure 7A), and even stronger inhibition was measured in separate experiments by titration of the fluorescent virus (Figure 7B). None of the other miRNAs tested in this study was found to have any measurable effect on RSV replication (data not shown).

## Discussion

This study shows for the first time that RSV infection of the human airway epithelium has profound effects on the pattern of miRNAs expression in host cells, with potentially important repercussions on the biosynthesis of key neurotrophic factors and receptors, programmed cell death, and viral replication. Of particular significance is the inhibitory effect exerted by this virus on miR-221, a miRNA predicted to target for degradation the mRNA transcripts encoding the anti-apoptotic neurotrophin NGF and its cognate high-affinity receptor TrKA. Accordingly, our data show that high intracellular levels of miR-221 reduce NGF expression, favor the apoptotic death of infected cells, and limit the infection significantly.

The model emerging from this study suggests that RSV removes a physiologic inhibitory control exerted by miR-221 on the NGF-TrKA pathway. By upregulating this pathway, the invading virus prevents apoptosis and keeps the infected cells alive to allow completion of its life cycle and establish lytic cycles of replication that result in propagation of the infection to the neighboring cells. Hence, our data propose miR-221 as an early regulator of RSV replication in the bronchial epithelium, upstream from the neurotrophic mechanisms described in our previous work [3]. As the transcripts for both NGF and TrKA were significantly reduced in cells overexpressing miR-221, it is more likely that these mRNA targets are primed for degradation, rather than translational inhibition. It also plausible that, in addition to the ones described in the present study, other mechanistic pathways might contribute to the control of neurotrophic pathways and modulation of virus-induced apoptosis. As an example, miR-331 slightly downregulated NGF protein levels, suggesting inhibition at the translational level; this may be evidence of a tandem downregulation of NGF by miR-221 transcriptionally and miR-331 translationally.

Although we were not able to procure cells from infants or young children for our *in vitro* studies, the present results are consistent and provide an explanation for the previously reported increase of NGF and TrKA expression in cells obtained by bronchoalveolar lavage (BAL) from human infants with RSV bronchiolitis [5]. Of importance, the information obtained with our *in vitro* experiments about the role of miRNAs in infected bronchial epithelial cells would be impossible to find using BAL or other clinical specimens, because the presence of extracellular miRNAs and multiple structural and inflammatory cell types from upper and lower airways, each having different miRNA expression patterns, would make it very difficult to identify the cellular origin and function of individual miRNAs.

### Neurotrophins

NGF is the prototypical member of a family of neurotrophic factors that includes the brain-derived neurotrophic factor (BDNF) and neurotrophins 3 and 4 (NT3 and NT4), and controls critical aspects of neuronal cells life like survival, proliferation, differentiation, neurite growth, and neurotransmission [14], [15]. Two types of membrane receptors mediate neurotrophic signaling: a) the three members of the TrK family of receptor tyrosine kinases TrKA, TrKB and TrKC, which exhibit selectivity for NGF, BDNF/NT4, and NT3, respectively; and b) the p75^NTR^ receptor, which is a member of the tumor necrosis factor (TNF) receptor superfamily and binds at low affinity all four neurotrophins, but can also bind at high affinity the NGF precursor (pro-NGF) and regulate the affinity of TrKA for its cognate ligand.

In addition to its neurotrophic functions, NGF participates in a number of other fundamental biological mechanisms that are highly preserved across species. In particular, by binding its TrKA receptor NGF modulates the activity of multiple non-neural cells involved in immune and inflammatory responses [2] and prevents apoptotic death of cells invaded by biological [3], [4] or physical [16], [17] agents. Therefore, the NGF-TrKA axis serves as an innate protective system that allows airway epithelial cells exposed to airborne threats to tolerate, and maybe even exploit, potentially pathogenetic agents. On the other hand, the anti-apoptotic activity of NGF may also, especially in conditions of relative immunological weakness, allow the persistence and/or propagation of the invading agent as shown with the human immunodeficiency virus (HIV) infection [4].

### MicroRNAs

Epigenetics was originally defined as “the causal interactions between genes and their products, which bring the phenotype into being” [18] and continues to evolve to include different patterns of DNA and RNA activity that do not depend on the genome nucleotide sequence, such as the regulation of gene expression by non-coding RNAs. Our data showing the involvement of miR-221 in the regulation of RSV replication provide the first evidence, to our knowledge, of an epigenetic mechanism capable of directing the fate of this highly prevalent respiratory infection.

MicroRNAs constitute a diverse class of small non-coding RNAs whose biogenesis starts with a large primary transcript (pri-miRNA), which is processed in the nucleus by a Drosha family member into a precursor hairpin (pre-miRNA) of 60–70 nucleotides. The Dicer enzyme further trims the precursor into a double-stranded RNA, typically 22 nucleotides in length. The functional strand from this duplex (mature miRNA) is incorporated into an RNA-induced silencing complex (RISC) that invariably contains a member of the Argonaute protein family. The active RISC is directed toward its mRNA targets to regulate, usually negatively, the translation of the message.

Each miRNA is able to target the translation of hundreds of partially complimentary mRNA transcripts, and can also alter gene transcription by targeting transcription factors and DNA methyltransferases. Therefore the resulting biological activity of individual miRNAs is not easily predictable. In fact, the complexity of this regulatory network may explain some unexpected results of our experiments, like a net increase in NGF expression following overexpression of a miRNA predicted to target the same gene (e.g., miR-574), or measurable inhibitory effects by miRNAs whose sequence shows no obvious homology with any neurotrophic gene (e.g., miR-331).

MiR-221 is highly conserved in vertebrates, is encoded on the same chromosome across species, and has a high degree of homology to the mRNAs encoding NGF (62%) and its TrKA receptor (68%). It has also been shown to be upregulated in some cancer cell lines [19] and to function as an oncogenic factor by targeting the cyclin-dependent kinase inhibitor p27Kip1 [20], [21], [22]; together, these observations suggest an important role of miR-221 in cell growth regulation. Based on our findings, we speculate that miR-221 may play a role in modulating severity and duration of acute bronchiolitis, and possibly its chronic sequelae like post-viral asthma. An important corollary of this hypothesis is that exogenous administration of synthetic miRNAs may antagonize RSV replication in its natural target, i.e., the distal airway epithelium, and therefore provide a novel strategy for the therapy of this common infection.

In this study, we focused our attention on miR-221 because: 1. It is significantly downregulated by RSV; 2. Its overexpression is associated with maximal downregulation of NGF and its TrKA receptor both at the mRNA and protein level; and 3. Cells transfected with this miRNA are less prone to RSV infection. However, our data also show significant RSV-induced changes in other miRNAs able to modify the expression of neurotrophic factors and receptors (e.g., miR-574, miR-453), which may also have biological significance and therapeutic potential. Furthermore, it is quite possible that other not yet identified or characterized miRNAs exist that either directly or via more complex epigenetic mechanisms affect neurotrophic pathways, programmed cell death and viral replication.

Depending on the kinetics of viral replication, miRNA patterns expressed by infected cells can influence the ability of the invading virus to replicate and spread [23]. On the other hand, several viruses (especially herpesviruses and other nuclear DNA viruses) also encode their own miRNAs that can alter or saturate the miRNA composition of host cells [24]. Thus, both the virus and the host are able to manipulate the miRNAome as part of their evolutionary strategies for survival [25], and in fact several studies have shown the involvement of both virus-encoded and cell-encoded miRNAs in prolonging host cell survival, evading immune recognition, and establishing viral latency [24], [26]. It should be noted, however, that the biosynthesis of virus-encoded miRNAs is virtually impossible in RSV-infected cells, as the life cycle of this virus is exclusively cytoplasmatic and implies lack of access to the nuclear Drosha enzymes [27].

The experimental strategy adopted in this study, involving the direct exposure of bronchial cells to mimic oligonucleotides - rather than antagomirs - to define the role of specific miRNAs in infected cells, was chosen because RSV itself acts like an antagomir, and therefore it would have been difficult to discern the individual effect of synthetic antagomirs in infected cells and dissect additive or synergistic interactions. A potential shortcoming of this approach, however, is that miRNA mimics are subject to off-target effects and the effect seen with a mimic can be increased as compared to an inhibitor.

Another important reason for using mimics was that we wanted to build on a recent randomized, double-blind, placebo-controlled clinical trial that demonstrated feasibility, safety, and potential efficacy of a small inhibitory RNA (siRNA) complimentary to the mRNA encoding the RSV N protein and delivered directly to the airway mucosa by nebulization [28]. The accompanying editorial listed among the limitation of this approach the specificity to a single viral target, with consequent lack of activity towards co-infecting pathogens, and the possible activation of innate immunity by exogenous siRNAs [29]. Both of these shortcomings could be addressed with the use of oligonucleotide mimic sequences of endogenous miRNA species physiologically involved in fundamental protective mechanisms like programmed cell death, which are essential for the innate defense of the host against a variety of invading pathogens.

In conclusions, our data show that RSV infection modifies miRNA expression patterns in its natural target, i.e., the human bronchial epithelium. In particular, this virus inhibits the endogenous expression of miR-221, which is highly complementary to the mRNAs encoding NGF and its cognate receptor TrKA and can target them for degradation. These observations suggest that, once the physiologic silencing of the anti-apoptotic NGF-TrKA axis is removed, the resulting inhibition of programmed death in the infected cells may favor lytic cycles of viral replication and spreading of infecting virions to neighboring cells. Thus, the present study provides the proof of concept that miRNAs are intimately involved in the intracellular mechanisms controlling RSV replication and can be manipulated pharmacologically for therapeutic purposes.
